# Viral quasispecies

**DOI:** 10.1371/journal.pgen.1008271

**Published:** 2019-10-17

**Authors:** Esteban Domingo, Celia Perales

**Affiliations:** 1 Centro de Biología Molecular Severo Ochoa (CSIC-UAM), Consejo Superior de Investigaciones Científicas (CSIC), Campus de Cantoblanco, Madrid, Spain; 2 Centro de Investigación Biomédica en Red de Enfermedades Hepáticas y Digestivas (CIBERehd) del Instituto de Salud Carlos III, Madrid, Spain; 3 Department of Clinical Microbiology, IIS-Fundación Jiménez Díaz, UAM, Madrid, Spain

## Abstract

**Viral quasispecies** refers to a population structure that consists of extremely large numbers of variant genomes, termed mutant spectra, mutant swarms or mutant clouds. Fueled by high mutation rates, mutants arise continually, and they change in relative frequency as viral replication proceeds. The term quasispecies was adopted from a theory of the origin of life in which primitive replicons) consisted of mutant distributions, as found experimentally with present day RNA viruses. The theory provided a new definition of wild type, and a conceptual framework for the interpretation of the adaptive potential of RNA viruses that contrasted with classical studies based on consensus sequences. Standard clonal analyses and deep sequencing methodologies have confirmed the presence of myriads of mutant genomes in viral populations, and their participation in adaptive processes. The quasispecies concept applies to any biological entity, but its impact is more evident when the genome size is limited and the mutation rate is high. This is the case of the RNA viruses, ubiquitous in our biosphere, and that comprise many important pathogens. In virology, quasispecies are defined as complex distributions of closely related variant genomes subjected to genetic variation, competition and selection, and that may act as a unit of selection. Despite being an integral part of their replication, high mutation rates have an upper limit compatible with inheritable information. Crossing such a limit leads to RNA virus extinction, a transition that is the basis of an antiviral design termed lethal mutagenesis.

## Historical origins

Quasispecies theory was developed in the 1970’s by Manfred Eigen and Peter Schuster to explain self-organization and adaptability of primitive replicons (we use the term replicon to refer to any replicating entity), as an ingredient of hypercyclic organizations that link genotypic and phenotypic information, as an essential step in the origin of life [1,2]. The theory portrayed early replicon populations as organized mutant spectra dominated by a master sequence, the one endowed with the highest fitness (replicative capacity) in the distribution. It introduced the notion of a mutant ensemble as a unit of selection, thus emphasizing the relevance of intra-population interactions to understand the response to selective constraints. One of its corollaries is the error threshold relationship, which marks the maximum mutation rate at which the master (or dominant) sequence can stabilize the mutant ensemble. Violation of the error threshold results in loss of dominance of the master sequence and drift of the population in sequence space) [2–5].

**Fig 1:**
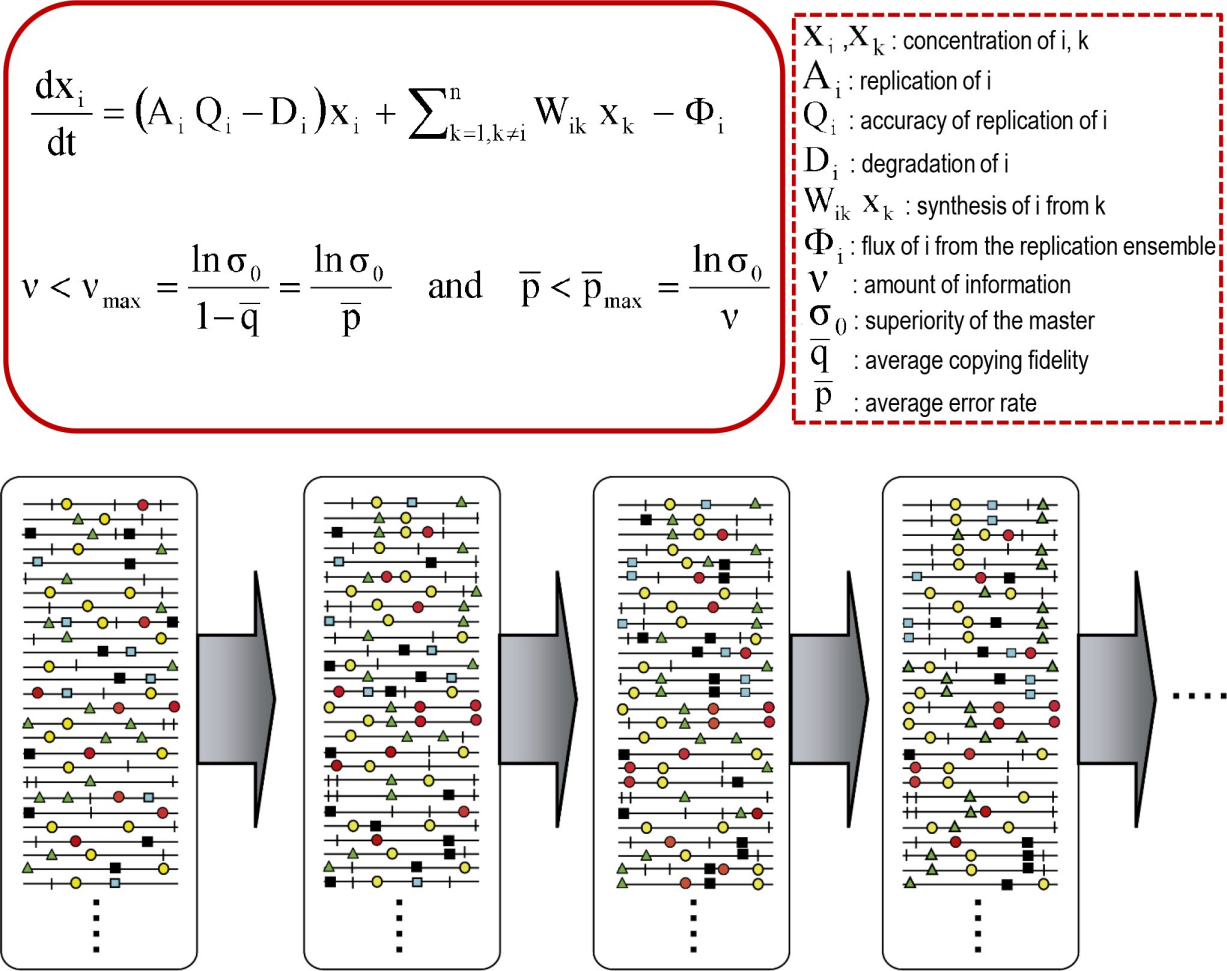
Fundamental equations of quasispecies and representation of mutant spectra. The equations are the mathematical expression of the major concepts implied by quasispecies theory. The first equation describes the change of concentration of molecule i as a function of replication parameters, and its production from other molecules of the same ensemble. The second equation is the error threshold relationship, indicating the maximum amount of information (ʋ_max_) and the maximum average error rate p_max_ (p = 1- q; q is the copying fidelity) for maintenance of genetic information. Terms are defined in the box on the right. Below, an evolving mutant spectrum (with mutations represented as symbols on the genomes), with an invariant consensus sequence. Details in [2].

**Fig 2:**
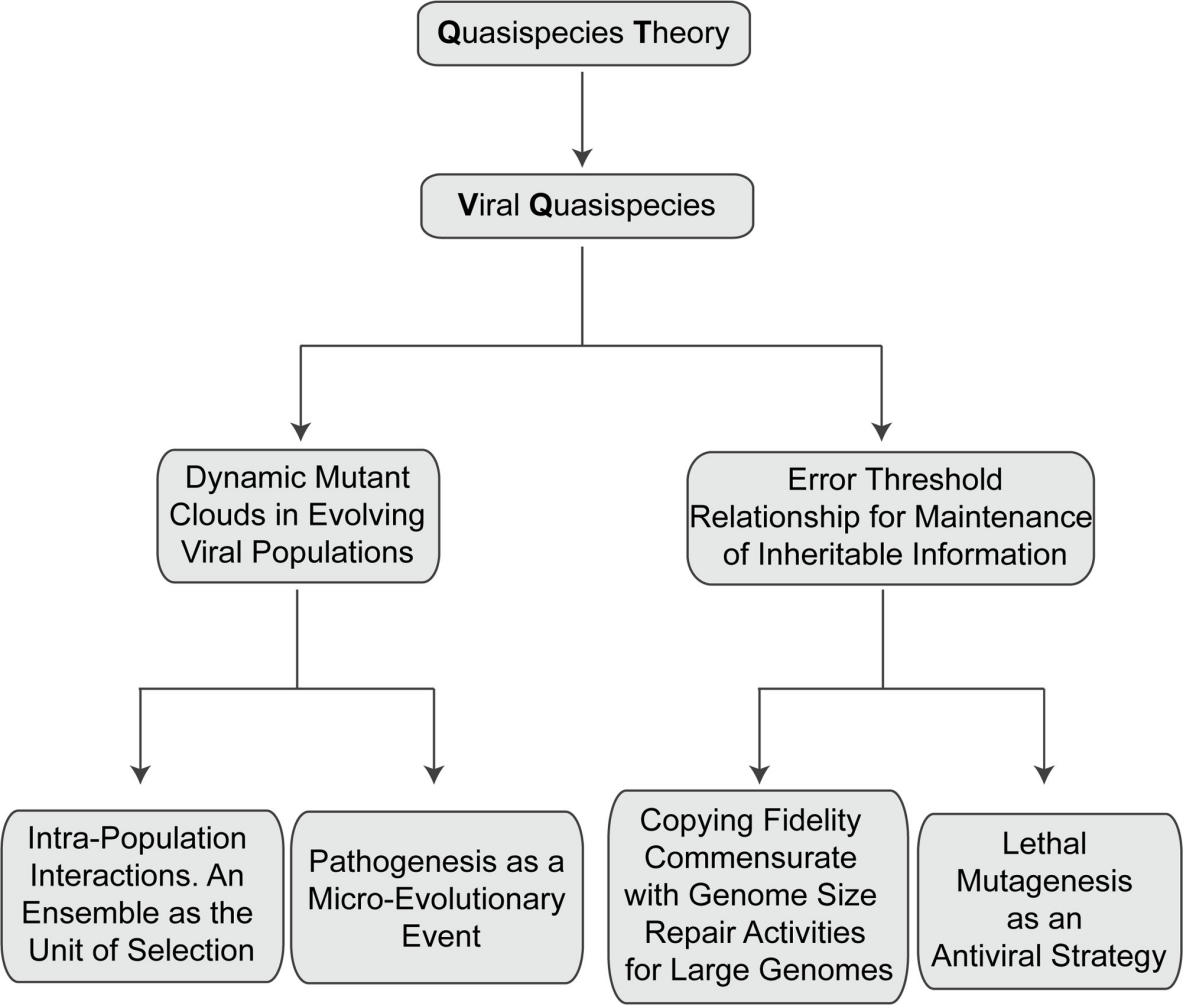
Flow of conceptual derivations of quasispecies theory for viral populations, and some biological consequences.

The core quasispecies concepts are described by two fundamental equations: replication with production of error copies, and the error threshold relationship (Fig 1). They capture two major features of RNA viruses at the population level: the presence of a mutant spectrum, and the adverse effect of an increase of mutation rate on virus survival, each with several derivations (Fig 2).

The existence of a mutant spectrum was experimentally evidenced first by clonal analyses of RNA bacteriophage Qβ populations whose replication had been initiated by a single virus particle. Individual genomes differed from the consensus sequence in an average of one to two mutations per individual genome [6]. Fitness of biological clones was inferior to that of the parental, uncloned population, a difference also documented for vesicular stomatitis virus (VSV) [7]. The replicative capacity of a population ensemble need not coincide with that of its individual components. The finding that a viral population was essentially a pool of mutants came at a time when mutations in general genetics were considered rare events, and virologists associated a viral genome with a defined nucleotide sequence, as still implied today in the contents of data banks [8]. The cloud nature of Qβ was understood as a consequence of its high mutation rate, calculated in 10^−4^ mutations introduced per nucleotide copied [9], together with tolerance of individual genomes to accept an undetermined proportion of the newly arising mutations, despite fitness costs. The error rate estimated for bacteriophage Qβ has been confirmed, and is comparable to values calculated for other RNA viruses [10,11].

High mutation rates and quasispecies were verified for other RNA viruses based on dissection of viral populations by molecular or biological cloning, and sequence analysis of individual clones. John Holland and colleagues were the first to recognize that a rapidly evolving RNA world inserted in a DNA-based biosphere had multiple evolutionary and medical implications [12–14]. Genome plasticity of RNA viruses had been suspected for many decades. Key early observations were variations in viral traits described by Findley in the 1930’s, the studies of Granoff on transitions of plaque morphology of Newcastle disease virus, or the high frequency of conversions between drug resistance and dependence in Coxsackie A9 virus, among other studies with animal and plant viruses in the middle of the 20^th^ century (for a historical overview and references, see 15). When put in the context of present day knowledge, we realize that these observations on phenotypic changes were the tip of the iceberg of an extremely complex reality of viral populations. High mutation rates and population heterogeneity characterize RNA viruses, with consequences for viral pathogenesis and the control of viral disease. Detailed studies on quasispecies dynamics *in vivo* have been performed with human immunodeficiency virus type 1 (HIV-1) and hepatitis C virus [16–18].

### Current scope of quasispecies

The first mathematical formulation of quasispecies was deterministic; it assumed steady state mutant distributions in equilibrium without perturbations derived from modifications of the environment or population size [2]. These conditions are common in initial theoretical formulations of complex phenomena because they confer mathematical tractability. Since then, several extensions of the theory to non-equilibrium conditions with stochastic components have been developed, with the aim of finding general solutions for multi-peak fitness landscapes. These objectives approximate quasispecies to the real case of RNA viruses, which are compelled to deal with dramatic variations in population size and environment (reviewed in [19]). Research on quasispecies has proceeded through several theoretical and experimental avenues that include continuing studies on evolutionary optimization and the origin of life, RNA-RNA interactions and replicator networks, the error threshold in variable fitness landscapes, consideration of chemical mutagenesis and proofreading mechanisms, evolution of tumor cells, bacterial populations or stem cells, chromosomal instability, drug resistance, and conformation distributions in prions (a class of proteins with conformation-dependent pathogenic potential; in this case the quasispecies is defined by a distribution of conformations) [16,20]. New inputs into experimental quasispecies research have come from deep sequencing to probe viral and cellular populations, recognition of interactions within mutant spectra, models of viral population dynamics related to disease progression and pathogen transmission, and new teachings from fidelity variants of viruses (the several theoretical, experimental and practical facets of quasispecies have been reviewed in several chapters of [20]). Here we summarize the main aspects of quasispecies dynamics, and recent developments relevant to virus evolution and pathogenesis.

## Dynamic heterogeneity

The molecular basis of high error rates is the limited template-copying fidelity of RNA-dependent RNA polymerases (RdRps) and RNA-dependent DNA polymerases (also termed reverse transcriptases, RTs). In addition, these enzymes are defective in proofreading) [21] because they lack a 3’ to 5’ exonuclease domain present in replicative cellular DNA polymerases [22]. Also, postreplicative-repair pathways, abundant to correct genetic lesions in replicating cellular DNA, appear as ineffective for double-stranded RNA or RNA-DNA hybrids. The presence of a proofreading-repair activity in coronaviruses increases their copying accuracy in about 15-fold [23]. This and other repair activities, that may act on standard RNA or retroviral genomes [24–27], do not prevent the formation of mutant spectra, although their amplitude may be lower than for other RNA viruses, at least in populations close to a clonal (single genome) origin. Quasispecies dynamics will operate in any viral or cellular system in which due to high mutation rates (as a result of low fidelity nucleic acid polymerases or environmental alterations) mutant spectra are rapidly generated [16,28–32].

Studies with different virus-host systems have established some general observations on the mechanisms of mutant generation, and implications of quasispecies dynamics [16,20,33–41]. In RNA virus genetics when we speak of “a mutant” the entity we handle is a cloud of mutants in which the specific mutation to which we direct our attention is present in all (or the great majority of) individual genomes. There is no such a thing as “a” wild type or “a” mutant virus. They are always clouds of mutants. Changes in the relative dominance of components of mutant spectra are particularly severe during *in vivo* infections, with complex dynamics of intra-host heterogeneity and variations. Bioinformatic procedures have been developed to unveil the relationships among different but closely related genome types that may suggest some hierarchical order of mutation acquisition or identification of transmission clusters (examples are **P**artition **A**nalysis of **Q**uasispecies, PAQ [42] or **QU**asispecies **E**volution, **N**etwork-based **T**ransmission **In**ference, QUENTIN [43]).

### Phenotypic reservoirs

The crux of the matter regarding quasispecies implications is that at any given time, the viral population includes a reservoir not only of genotypic but also of phenotypic variants, conferring upon the population some adaptive pluripotency. Accumulating laboratory and clinical evidence renders untenable that minority components of mutant spectra should be dismissed on the grounds of their being neutral. They can participate in selective processes and cannot be excluded from interpretations of virus behavior. Variation universally involves point mutations and it can also include recombination (in its replicative and non-replicative modes), and genome segment reassortment [33]. All modes of molecular variation are compatible, only restricted by the scope of mechanisms accessible to the replicative machinery, and for the need of viral genomes to remain functional. David Evans and colleagues identified many recombination events associated with enterovirus replication, and only a few recombinants made their way towards continued replication [44]. Recombination can mediate adaptability and virulence [45]. High mutation and recombination rates have led to the conceptual distinction between mechanistically unavoidable and evolutionarily relevant variation, in connection with the issue of clonal versus non-clonal nature of virus evolution (microbial evolution in general) [46,47]. Only a minority of the nascent variation during replication can be successfully propagated. Within limits that are set by biological constraints, each population is made of an array of variant genomes, with a total number which is commensurate with the virus population size. To infect a plant, animal or cell culture with 10^3^ infectious units can have very different consequences than to infect with 10^10^ infectious units, not only because the host defense systems may be overwhelmed by the high infectious dose, but also because the mutant repertoire that engages in adaptive explorations is larger. Part of the variants of a mutant spectrum, either in isolation or in consortium with others [48], may perform better than other members of the same population in the event of an environmental change. Selective pressures favor replication of some components of a mutant spectrum over others, despite all of them being interconnected by mutation. Differential performance can be at the level of viral genomes (during replication, intracellular gene expression, interaction with host factors, etc.) or viral particles (for thermal stability, entry into or exit from cells, to withstand neutralizing antibodies, etc.) [14,16–18,20,33–35].

Adaptability of RNA viruses is linked to parameters that facilitate exploration of sequence space: genome size (1.8 to 33 Kb), population size (variable but that can attain an impressive 10^12^ individual genomes in an infected host at a given time), replication rate, mutation rate, fecundity (yield of viral particles per cell), and number of mutations required for a phenotypic change (surprisingly low for several relevant traits) (see [49]).

**Fig 3:**
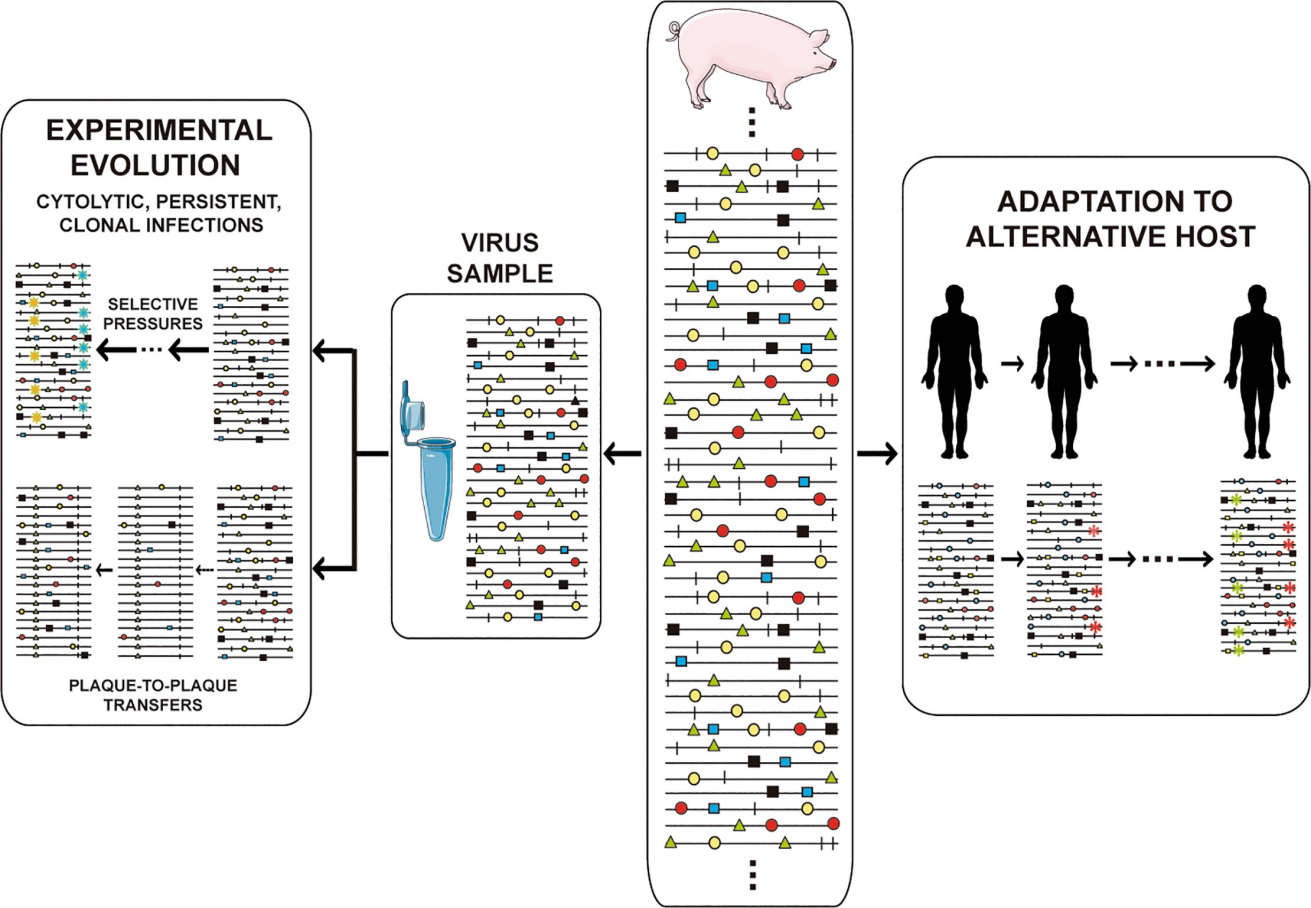
Scope of viral population dynamics. Upon isolation from an infected host (middle boxes), a virus sample may be adapted to cultured cells and subjected to large population or bottleneck transfers (left box), or be adapted to a different host *in vivo* (right box). Relevant adaptive mutations are highlighted with colored symbols.

Mutant spectrum dynamics has been depicted in different ways, and we have chosen one that encompasses frequent events in natural populations and research designs, such as virus isolation from an infected host, adaptation to cell culture for studies on experimental evolution, or adaptation to alternative hosts in vivo (Fig 3). Despite the complexity conveyed by the figure, the reality is even more complex, given the large population sizes, with an indeterminate proportion of genomes actively replicating at any given time (sometimes equated with the effective population size in general genetics), and harboring multiple mutations per genome. The scenarios suggested by current experimental data defy our imagination. The relative frequency of individual mutations fluctuates in an unceasing exploration of sequence space [50–52], with phenotypic changes (not only genotypic changes) being far more frequent than previously thought. The experimental evolution design that consists of passaging viral populations for long time periods (many sequential infections) is often extremely revealing. In foot-and-mouth disease virus (FMDV) such a design led to a remarkable phenotypic diversification into subpopulations of colonizers and competitors, that modulated virulence of the mutant ensemble [53]. In HCV such a design unveiled continuous mutation waves and a more accurate understanding of the types of fitness landscapes occupied by high fitness viruses [51,54].

### Limitations and indeterminacies

The nucleotide sequence of an individual genome from a population (no matter which the degree of population complexity might be), can be determined either following a biological or molecular cloning event or by deep sequencing of entire viral genomes, in a manner that mutation linkage (assignment of different mutations to the same genome molecule) can be established. Each of these procedures implies some limitations: biological cloning can bias the representation in favor of infectious genomes, while molecular cloning can introduce non-infectious (defective) genomes in the analysis [14,49,50]. Whole genome quasispecies description is still technically challenging due to the artifactual introduction of mutations. Most current deep sequencing platforms yield sequences of short reads for a given amplicon (sequence under analysis); minority mutations in an amplicon cannot be reliably linked to mutations in a different amplicon of the same genome; at most, statistical inferences on linkage can be proposed. Despite these limitations, control experiments and improvements of bioinformatic procedures support that the majority of sequence heterogeneity analyzed in viral populations indeed reflects differences in the natural template populations. If mutation linkage can be solved on a routine basis, a new wave of molecular information relevant to epistatic interactions will enter the picture.

There are additional levels of indeterminacy in the sequential analysis of viral populations, in particular those replicating in vivo. Components of the mutant spectrum represented at a given time in the sample taken for sequencing may differ from those in the next time point, due either to sampling uncertainties or bona fide fluctuations of genome frequencies. It is not justified to accept a rough similarity because even a single mutation in a given sequence context may affect biological properties [16]. In the words of John Holland and colleagues: “It is important to remember that every quasispecies genome swarm in an infected individual is unique and “new” in the sense that no identical population of genomes has ever existed before and none such will ever exist again” [55]. On top of the fleeting nature of any mutant distribution, the standard methods available for quasispecies characterization provide genomic sequences of a minority of the population (estimated in 10^−8^ to 10^−13^ for molecular cloning-Sanger sequencing, and in 10^−6^ to 10^−11^ for deep sequencing; reviewed in [49]). We can only have an approximate representation of viral populations and their dynamics, as evidenced by many experimental studies [6,12,16,34,35,49,51,56].

### Consensus is not enough

The points summarized in previous sections fully justifies addressing analytical tools towards the mutant spectrum rather than ignoring it or considering its presence a side issue. Use of consensus sequences to describe the genome of a virus isolate, despite being warranted by the difficulties of conveying the information recapitulated in a mutant spectrum, blurs and enfeebles biological interpretations. Experimental results have demonstrated that minority genomes from a mutant spectrum (that cannot be identified by examining the consensus sequence) can include mutations that confer resistance to antiviral inhibitors, neutralizing antibodies or cytotoxic T cells, or that can alter the capacity to induce interferon (IFN) or to respond to IFN, virulence or particle stability, among other phenotypic traits [16,33,37,51,56–60]. Mutant spectra can also mediate cyclical adaptation to different cell types [61]. A mutant spectrum defines a consensus but the consensus is an abstraction; it may not be represented in the population. Many events in viral pathogenesis and evolution are due to mutant spectrum modifications or interactions which cannot be properly interpreted solely on the basis of consensus sequences [6,14–16,20,32,34,35,44,45,48,51,55].

## Collective responses: ensembles as units of selection

Mutant spectra are not mere aggregates of mutants acting independently. They are often engaged in collective responses of two major types: those that depend on the presence of sets of variants, and those that rely on intra-mutant spectrum interactions.

### Variants that drive responses to selective constraints

#### Behavior of reconstructed quasispecies

In some cases of sweeping selection (very strong selection for a trait), an individual (or a limited number of individuals) that encodes signatures prone to be selected, may approach dominance while becoming the founder of a mutant cloud (because formation of a cloud is inherent to replication). Conditions for dominance (in this case in response to selection) are that the genome senses the selective sweep and that its replication in the new selective environment is permitted. In other cases, a collection of mutants is selected. This was illustrated with a FMDV quasispecies that was reconstructed in the laboratory with multiple antigenic variants (each at low frequency) that belonged to two different categories, and shared resistance to the same monoclonal antibody [62]. One category included mutants with an amino acid substitution that affected receptor recognition (since the antigenic determinant overlapped with the integrin receptor recognition site); in the other category, the substitutions affected the antigenic determinant but not the receptor recognition site. Passages of the virus in absence of the monoclonal antibody resulted in dominance of antigenic variants that maintained the receptor recognition capacity, but the dominant variants were surrounded by a cloud of mutants of the other antigenic variant category. Conversely, passages in the presence of the antibody led to selection of variants with altered receptor recognition, surrounded by a cloud of antigenic variants that maintained receptor recognition. The results underlined the role of mutant clouds in selective events, and unveiled a new mechanism of antigenic flexibility [62].

#### Quasispecies memory

Quasispecies memory is a type of molecular memory dependent on the recent history of the evolutionary lineage and the integrity of the mutant spectrum [57,63]. The search for memory was prompted by the complex adaptive system behavior of a viral quasispecies, suggested by the presence of core information (considered the one that defines viral identity) despite variation of constitutive elements (the mutant spectrum). A well-known example is memory in the immune system that mobilizes and expands minority components in response to stimuli previously faced by the system [64]. In the experiments designed to identify memory in viral quasispecies, members of the mutant spectrum increased in frequency as a consequence of their replication during a selection event that drove them towards dominance. When the selective constraint was withdrawn, memory genomes remained at levels that were 10- to 100-fold higher than the basal levels attributable solely to their generation by mutation, as documented with independent FMDV genetic markers, and with HIV-1 *in vivo* [57,63,65,66]. Thus, memory is a history-dependent, collective property of the quasispecies that confers a selective advantage to respond to environmental changes previously experienced by the same evolutionary lineage. It can be manifested only if the mutant spectrum maintains its completeness, since memory is lost when the population undergoes a bottleneck event that excludes minorities. A relevant example of the consequences of memory occurs in antiviral pharmacology with the administration for a second time of the same or a related antiviral agent (capable of evoking shared resistance mutations) used in a previous treatment. The second intervention may face inhibitor-resistant memory genomes from the earlier treatment, thus contributing to virus escape [57]. This is an aspect that has not received adequate attention in the planning of antiviral interventions for patients who fail a first treatment and have to be subjected to a second treatment.

### Intra-mutant spectrum interactions for interference, complementation or cooperation

**Individual genomes** surrounded by a cloud of related mutants can be either suppressed to be kept at low frequency, or helped to be maintained in the population. The two alternative fates are dependent on several factors, one being the surrounding mutant spectrum in those steps of the infectious cycle in which an effective competition among variants is established, for example within replication complexes. This important concept was first derived theoretically [3,67], and then approached experimentally with several viruses. In an early study, Juan Carlos de la Torre and John Holland described suppression of high fitness VSV by mutant spectra of inferior fitness [68]. Suppressive effects have since been documented with standard and mutagenized viral populations. Some examples are:

- Suppression of high fitness antigenic variants of FMDV by low fitness antibody-escape mutants [69].
- ‏Suppression of virulent poliovirus (PV) by attenuated virus in poliovirus vaccines [58].
- Suppression of pathogenic lymphocytic choriomengitis virus (LCMV) (that cause growth hormone deficiency in mice) by non-pathogenic LCMV variants [70].
- Suppression of FMDV by a mutagenized FMDV population [71].
- Suppression of FMDV by capsid and polymerase FMDV mutants [72].
- Suppression of drug-resistant viral mutants during antiviral therapy [73,74].

Opposite to suppression is maintenance of a mutant either by a favorable position in a fitness landscape or by interactions of complementation or cooperation with members of the mutant spectrum. The position in a fitness landscape influences vulnerability to mutations, as popularized with the terms “advantage of the flattest” or “survival of the flattest”, indicating that a variant located at the top of a sharp fitness peak has higher probability to decrease fitness as a result of new mutations than the same variant located at a fitness plateau [75–77]. Survival of the flattest has been also proposed as an ingredient in some models of the error threshold [78].

Collective behavior of viruses was documented with mutant RNA viruses resistant to nucleotide analogues. The study of this class of mutants has been instrumental for the understanding of the molecular basis of template copying fidelity, and the consequences of fidelity alterations in the adaptive capacity and pathogenic potential of RNA viruses [79–81]. In the first mutant studied, amino acid substitution G46S in the PV polymerase resulted in about four-fold increase in template-copying fidelity. This modification reduced PV adaptability and infective potential *in vivo* [79,80]. The mutant in isolation did not replicate efficiently in the brain of susceptible mice, but it did when its mutant spectrum was broadened by 5-fluorouracil mutagenesis or when it was co-inoculated with wild type PV [80].

Complementation (often occurring when a functional protein encoded by a set of genomes is used by another set of genomes whose encoded protein is not functional) may underlie some collective responses of quasispecies such as fitness of individuals isolated from a population being inferior to fitness of the population [6,7]. Complementation was described between two truncated FMDV genomic forms [56,82]. The genomes with internal deletions became detectable upon high multiplicity passage of a clonal population of standard FMDV, a virus with a monopartite single stranded RNA genome. Infectivity was generated by complementation of the two truncated forms, in absence of standard, full length FMDV genomes. For complementation to be effective, prior exploration of sequence space through point mutations was a requirement [83]. The system underwent a remarkable evolutionary transition akin to genome segmentation. Drastic genetic lesions in viral genomes are difficult to observe unless a mechanism such as complementation comes into the rescue of the deviant genomes. Additional examples of complementation among RNA viruses have been reported ([84–86]; for review see [33,35]). Complementation is a means to maintain defective genomes at detectable frequencies in viral populations.

A distinction has been made between complementation and cooperation, in which two different genomes give rise to a new phenotype through the interaction between two variant proteins [87]. An example of cooperation was characterized during studies with measles virus on membrane fusion which is essential for virus entry into cells. For this virus fusion is mediated by two proteins termed H and F. A truncated H was deficient in cell fusion but the activity was regained when the truncated H was accompanied by two forms of F but not one of the forms individually [87].

Therefore, complementation, cooperation, interference and suppression can emerge from interactions among components of mutant spectra that have their origin in random mutations. Selection acts on whatever sets of mutants can provide a useful trait, to turn random occurrences into biological meaning.

## Bottlenecks: Weakening, liberation, or challenge?

**Fig 4:**
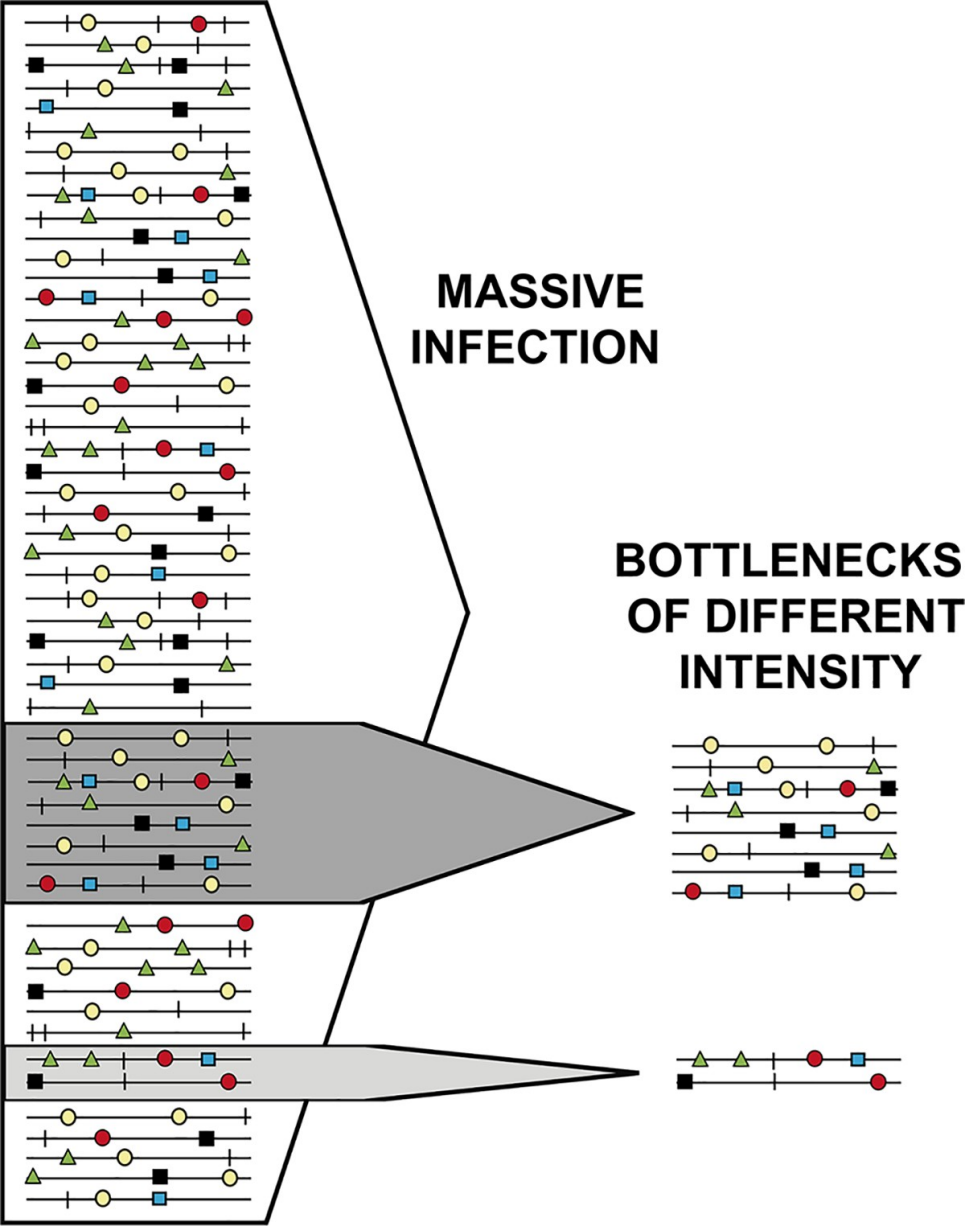
Illustration of bottleneck of different severity, defined by the different arrows acting on the entire population. Symbols represent mutation types.

A means to interrupt the participation of individual genomes in interactions with their mutant spectrum is for the quasispecies swarm to undergo drastic reductions in population size that isolate one or few individual genomes from their surroundings. Such reductions are termed bottlenecks (Fig 4), and they have an important participation in shaping evolutionary lineages for all kinds of organisms, and also for viruses. Bottleneck events are also depicted in Fig 3 (plaque-to-plaque transfers in box at the left). They occur frequently not only upon host-to host transmission but also inside infected hosts [88–90], and they can perturb positive and negative selection events in processes that are difficult to identify and characterize.

Drastic bottleneck events have been reproduced with laboratory populations of viruses in the form of plaque-to-plaque transfers [91,92] (depicted in Fig 3). This design served to verify experimentally the operation of Müller’s ratchet, or fitness decrease by the irreversible incorporation of mutations in asexual organisms in absence of compensatory mechanisms [93]. The serial bottleneck transfers unveiled the presence of rare mutations, not seen in standard laboratory or natural viral populations. In absence of forced bottleneck events, such rare mutations would be lost by negative selection because of the fitness cost they inflict [94]. The investigation of how FMDV clones debilitated by Müller’s ratchet regained replicative fitness revealed several alternative molecular pathways for fitness recovery [95]. The implications of this observation went largely unnoticed until recent results with hepatitis C virus (HCV) have also suggested the accessibility of multiple pathways for fitness gain [51,54]. Also, extensive passage of a biological clone of FMDV in BHK-21 cells conferred the capacity to infect several human cell lines in addition to the expected fitness increase for multiplication in BHK-21 cells [96]. Thus, several lines of evidence suggest that fitness gain in a specific environment may paradoxically broaden the phenotypic potential of a virus. It will be interesting to investigate whether focused adaptation of other viruses to a specific environment may also entail a broadening of diversity, with many phenotypic variants attaining similar fitness levels. If generalized, this broadening of phenotypic space would provide a new interpretation of the molecular basis of adaptation, and explain why adaptation to alternative environments may not lead to attenuation.

Deprivation of an individual virus from possible suppression, complementation or cooperation, may represent a liberation to initiate a new evolutionary process, or a condemnation to extinction. If liberated from suppression, the isolated genome must replicate and be able to reconstruct a mutant cloud to regain adaptive capability. This has led to the suggestion that high mutation rates evolved to allow such mutant spectrum recovery following bottlenecks. Other models attribute high mutation rates to adaptive optimization independent of bottlenecks, or to a mechanistic consequence of rapid replication (reviewed in [49]). Whatever their ultimate origins, high mutation rates serve the purpose of adaptation in multiple circumstances, not only following bottlenecks. A founder virus can introduce a different phenotype for the ensuing evolution. Evolution of viruses in nature and as disease agents can be viewed as succession of mutant spectrum alterations, subjected to expansions and reductions of population size in a continuous interplay of positive and negative selection and random drift. While short-term (for example, intra-host) evolution is observable and measurable, viruses may appear to be relatively static in the long term for decades (as seen with antigenic variants of FMDV [97]) or longer. Intra-host evolution is generally more rapid than inter-host evolution, as documented with viruses [16] and other biological systems [98]. Apparent invariance may be the result of selection for long-term survival of populations that have previously frenziedly tested evolutionary outcomes in short-term processes [49].

## Quasispecies and viral disease

Soon after quasispecies was evidenced for viruses, some medical implications were made explicit [12,99]. Several specific or general points (reviewed in [16,34,59], and in several chapters of [20]) can be succinctly exposed as follows:

- High mutation rates and population heterogeneity endow viruses with the potential to escape immune pressures (including those due to vaccination) and antiviral inhibitors used in therapy. It is an open question if vaccination can promote long-term evolution of antigenic determinants.
- Attenuated RNA virus vaccines can revert to virulent forms. RNA viruses released in nature for pest control purposes can mutate to new phenotypes.
- Virus attenuation and virulence is dependent on viral genetic traits. Variant forms of a given virus may display increased virulence or atypical disease.
- Components of a mutant spectrum can exhibit a different cell tropism or host range than most genomes in the same population, with implications for the emergence and re-emergence of viral disease.
- Viral pathogenesis is influenced by microevolutionary processes in which some viral subpopulations are replaced by others to persist or to invade new cell types, tissues or organs.
- The larger the actively replicating (effective) population size and the replication rate, the most effective is exploration of sequence space for phenotypic expansions that favor survival and persistence.
- There is a connection between four parameters that caracterize viruses during infection processes: replication rate (the rate at which viral RNA or DNA is synthesized intracellularly for viral progeny production), viral load (the total amount of virus quantified in an infected host or host compartment), genetic heterogeneity, and replicative fitness (the yield of infectious particles that can contribute to the next generation). They can influence disease progression, and any of them can be targetted for disease control.

In all interactions conductive to disease, the host cells individually and as groups in tissues and organs play decisive roles. The consequences of a viral infection are always host-dependent. However, the virus itself poses a major challenge that a deeper understanding of quasispecies dynamics is helping to confront.

## Antiviral strategies in response to the quasispecies challenge

There is an increasing perception that Darwinian principles should assist in the planning of antiviral designs [100]. The aim of vaccination is to evoke a protective response that either prevents virus replication or disease. The aim of an antiviral pharmacological intervention is to inhibit virus replication to provide the immune system with an opportunity to clear the virus. Expressed simply, the direct danger for vaccination and treatment is that virus can escape through selection of mutants resistant to vaccine-triggered defense components or to the externally administered inhibitors. This has led to several proposals to confront viral disease, that can be summarized as follows (reviewed in [49]):

### Vaccines should expose multiple B cell and T cell epitopes to the immune system

Vaccines should include repertoires of B cell and T cell epitopes to evoke an ample immune response. The broad response should minimize selection of escape mutants that may be present as minority components in mutant spectra, as repeatedly documented experimentally [14,16,35,57]. With the current types of available vaccines, those that best comply with the multiple epitope requirement are, in the order of expected efficacy to confer protection against highly variable viruses: attenuated > inactivated whole virus > several expressed proteins > one expressed protein > multiple synthetic peptide antigens > single peptide antigen. The scarcity of effective synthetic vaccines for RNA viral pathogens despite huge scientific and economic efforts is a reflection of the underlying problems.

### Antiviral agents should be used in combination

Antiviral monotherapy (use of a single antiviral agent) is to be avoided. The following recommendations have been made and in some cases successfully implemented:

- Inhibitors used in combination should target different viral gene products.
- Splitting a treatment into two steps: first an induction regimen, and a second maintenance regimen. Drugs administered in the two steps should be different.
- Targetting of cellular functions needed for the virus life cycle.
- Use of innate immune response-stimulating drugs (for example, inhibitors of enzymes involved in pyrimidine biosynthesis).
- Combined use of immunotherapy and chemotherapy.
- Lethal mutagenesis or virus extinction by excess of mutations introduced during viral replication.

These strategies (whose supportive theoretical and experimental evidence has been reviewed in [49,60]) have as their main objective to avoid selection of treatment-escape mutants by multiple selective constraints that cannot be surmounted by the virus. Control is effective either because exploration of sequence space cannot reach the required multiple mutations (even when recombination is available) or because the multiple mutations inflict a severe fitness cost [101]. Vaccines exposing multiple epitopes and combination therapies follow the same strategy whose aim is to limit possible escape routes to viral quasispecies in the face of the suppressive constraint.

### Lethal mutagenesis

Lethal mutagenesis is the process of virus extinction at the error rate at which a virus can no longer maintain its genetic information [16,20,35,49,54,78,102,103]. Application of lethal mutagenesis as an antiviral strategy deserves attention in the context of the present article because its origins lie in quasispecies theory, in the form of the error threshold relationship (Fig 1). Both the error threshold and lethal mutagenesis are highly fitness landscape-dependent, but both can occur in complex fitness landscapes as those pertinent to viral populations [5]. The term lethal mutagenesis was coined by Lawerence Loeb and colleagues [102], and it is now widely used to describe the antiviral activity of base and nucleoside analogues that increase the viral mutation rate. Although several models have been proposed to account for virus extinction by excess mutations [78], an extension of the violation of the error threshold stands as a likely mechanism ([104]; recent review in [103]). Interestingly, some antiviral agents licensed for human use, initially thought to act only as inhibitors of viral replication, may actually exert their antviral activity against some RNA viruses at least partially by lethal mutagenesis. This is the case of favipiravir (T-705; 6-fluoro-3-hydroxy-2-pirazinecarboxamide) and ribavirin (1-β-D-ribofuranosyl-1-H-1,2,4-triazole-3-carboxamide) that are currently being intensively investigated as lethal mutagens [103].

Defense mechanisms based on genome modification of invading genetic parasites such as editing cellular activities that are recruited as part of the innate immune response (ADAR, APOBEC, RIP, etc; reviewed in [105]) represent a natural counterpart of the principle utilized by lethal mutagenesis. Applicability to pathogenic cellular elements is a real possibility, and lethal mutagenesis to control tumor cells is an active field of investigation [106,107]. Thus, the recognition of quasispecies dynamics has suggested some fundamental guidelines for disease prevention and control that are gradually permeating clinical practice. This is in line with the recognized need to apply Darwinian principles to the control of infectious disease.

## Summary and prospects

The main concepts covered in the present article and their domains of applicability are summarized in Table 1. The adequacy of quasispecies theory (versus other formulations of evolutionary dynamics [108]) as a framework for the error-prone replication of viruses and its consequences stems from its including mutation as an integral part of the replication process [13]. Quasispecies dynamics poses a great challenge for the molecular interpretation of short-term evolutionary events that bear on virus-host interactions and disease processes. A rewarding aspect of progress in having captured the meaning of the challenge (at least partially) is that we can exclude some of the prevention and control strategies that once were considered an option. A significant example is the historical rejection of combination therapies as contrary to the established canons of pharmacology, while now monotherapy is considered a risky practice (for discussion of this point, see [49]). We understand now that focused antiviral barriers that involve a single constraint (one inhibitor, one monoclonal antibody, one peptide antigen as vaccine) have a large probability of failing. Likewise, attempts to produce “universal” drugs, vaccines or diagnostic tools are unlikely to succeed given the intra-population diversity (present and potential) of the pathogenic agents to be controlled. A viral genome region which is conserved among types, subtypes, isolates may be so only regarding a consensus sequence but not the underlying mutant spectrum. Methods are now available to identify low level mutations that may predict escape from selective constraints. On a related note, it remains extremely unlikely to predict the emergence and re-emergence of viral diseases for a number of interconnected reasons, not the least important being pathogen adaptability [109]. The outlined limitations to predict the occurrence of viral diseases and to control them are based on our current understanding of viral complexity and dynamic change of such complexity. Obviously, we may be wrong, and in science we are open to the unexpected.

**Table 1:** Summary of main concepts related to quasispecies and their implications a.

Concept	Implications for virology	References
**Error-prone replication and mutant spectra**	Limited template-copying fidelity leads to formation of dynamic mutant distributions. They mediate virus adaptability.	[1,6,9,13]
**Phenotypic reservoir**	Mutant spectra are a phenotypic reservoir for selection to act upon	[16,17,20]
**Adaptive parameters**	Viral quasispecies adaptability relies on six parameters: genome size; population size; replication rate; mutation rate; fecundity; and number of mutations required for a phenotypic change	[16,35,49]
**Intra-mutant spectrum interactions**	Mutant spectra are not mere mutant aggregates. Emergent behavior can result from positive interactions of cooperation or complementation or negative interactions of interference among components cf the mutant spectrum	[6,7,68–70,82,85–87]
**Quasispecies memory**	A record of past genome dominances that prepares a viral population to respond to a selective constraint previously experienced by the same lineage. Bottlenecks erase quasispecies memory.	[57,63,65,66]
**Sequence space**	The total number of genomic sequences available to a virus. Adaptation is a movement towards a favorable region of sequence space. De-adaptation (i.e. lethal mutagenesis) is a movement towards unfavorable regions of sequence space.	[2,14,37,49]
**Population bottleneck**	A drastic reduction in population size. It promotes random drift in evolutionary outcomes. The diversifying effect of bottlenecks is accentuated by the cloud nature of viral populations.	[88,90,91,94]
**Biological constraints**	High mutation rates expand sequence space occupation. Biological constraints impose negative selection on many newly generated mutants. Constraints contribute to maintenance of virus identity.	[6,16,33]
**Connections with viral pathogenesis**	Progress of an infection is often associated with virus adaptation to host environments. Variants of the same virus can differ in disease potential (virulence).	[16,18,49]
**Quasispecies and long-term evolution**	Short-term evolutionary rate based on reorganization of mutant spectra is faster than long-term evolutionary rate. Conceptual links between quasispecies and phylodynamics at the epidemiological level are needed.	[111]

aConcepts are listed in the order relevant to the topics covered in the text, and serve to underline key points and some supportive studies. The concepts are expanded in the text, with additional references.

Quasispecies poses also a challenge for the annotation of the events we witness. Reality is far more complex than the means we have developed to describe viral populations in continuous change. How could the problem we approached? Procedures to organize and relate sequences for phylodynamic purposes need updating to exploit increased computing power to handle mutant spectra rather than one or few (consensus or other) sequences from each biological sample. The expanding number of sequences expected to become available including complete genomes (that will allow mutation linkage to enter the picture) if handled properly, may inform of the molecular basis of new phenotypes. The penetration into mutant spectra has also modified the concept of rare mutation since what in terms of consensus could be catalogued as rare may in fact be frequently occurring but rarely observed. A mutation which has low frequency at one time point or in a given environment may rise to high frequency at another time point or in a different environment. Rare mutations that populate mutant swarms may belong to defective genomes exerting relevant host interactions [110]. On a more general note, recognition of viral quasispecies was premonitory of the impressive diversity in the biological world that the metagenomic approaches are currently unveiling, including cellular heterogeneities whose biological implications remain largely unexplored.

## Supporting information

S1 Text Version history of the text file. (XML)

S2 Text Peer reviews and response to reviews. (XML)
